# Supplementary material for: Mapping the evolution of fertility support policies in China: A content and instrumental analysis
Source: PLoS One. 2025 Oct 9;20(10):e0332137. doi: 10.1371/journal.pone.0332137 (PMC12510515; doi:10.1371/journal.pone.0332137)
Supplement: S1 Appendix — (ZIP) [file pone.0332137.s001.zip › S1 Appendix. 226 original policy documents/196-国家发展改革委、国家卫生健康委关于印发《支持社会力量发展普惠托育服务专项行动实施方案(试行)》的通知(附 支持社会力量发展普惠托育服务专项行动项目和资金管理办法(试行))(FBM-CLI-4-336794).doc]

国家发展改革委、国家卫生健康委关于印发《支持社会力量发展普惠托育服务专项行动实施方案(试行)》的通知(附：支持社会力量发展普惠托育服务专项行动项目和资金管理办法(试行))

**发布部门：** [国家发展和改革委员会(含原国家发展计划委员会、原国家计划委员会)](https://www.pkulaw.com/law/chl?Aggs.IssueDepartment=60301) [国家卫生健康委员会](https://www.pkulaw.com/law/chl?Aggs.IssueDepartment=60321) [机构沿革](javascript:void(0))

**发文字号：**发改社会〔2019〕1606号

**发布日期：**2019.10.09

**实施日期：**2019.10.09

**时效性：** [现行有效](https://www.pkulaw.com/law/chl?Aggs.TimelinessDic=01)

**效力级别：** [部门规范性文件](https://www.pkulaw.com/law/chl?Aggs.EffectivenessDic=XE0302)

**法规类别：** [社会福利与社会保障](https://www.pkulaw.com/law/chl?Aggs.Category=01505)

国家发展改革委、国家卫生健康委关于印发《支持社会力量发展普惠托育服务专项行动实施方案（试行）》的通知
（发改社会〔2019〕1606号）

各省、自治区、直辖市及计划单列市、新疆生产建设兵团、黑龙江省农垦总局发展改革委、卫生健康委：
　　为深入学习贯彻习近平新时代中国特色社会主义思想和党的十九大精神，落实政府工作报告任务要求，深入实施《[国务院办公厅关于促进3岁以下婴幼儿照护服务发展的指导意见](https://www.pkulaw.com/chl/434b24b8b5769383bdfb.html)》，激发社会力量参与积极性，着力增加3岁以下婴幼儿普惠性托育服务有效供给，国家发展改革委、国家卫生健康委共同起草形成了《支持社会力量发展普惠托育服务专项行动实施方案（试行）》（以下简称《实施方案》）。
　　现印发给你们，请各地按照《实施方案》及附件《支持社会力量发展普惠托育服务专项行动项目和资金管理办法（试行）》要求，在教育现代化推进工程专项下，认真做好项目前期准备工作，加强对《实施方案》实施的监督检查，确保建设质量，提高资金使用效益。

国家发展改革委
国家卫生健康委
2019年10月9日

　　支持社会力量发展普惠托育服务专项行动实施方案（试行）

　　为深入学习贯彻习近平新时代中国特色社会主义思想和党的十九大精神，落实政府工作报告任务要求，深入实施《[国务院办公厅关于促进3岁以下婴幼儿照护服务发展的指导意见](https://www.pkulaw.com/chl/434b24b8b5769383bdfb.html)》，充分发挥中央预算内投资示范带动作用和地方政府引导作用，激发社会力量参与积极性，着力增加3岁以下婴幼儿普惠性托育服务有效供给，拟在全国开展支持社会力量发展普惠托育服务专项行动，制定本方案。
　　**一、**总体思路
　　3岁以下托育服务属于非基本公共服务范围，是地方政府事权，要坚持社会化发展托育服务，围绕“政府引导、多方参与、社会运营、普惠可及”，深入开展城企合作。国家通过中央预算内投资，支持和引导城市政府（包括设区市、自治州和县（市、区）等，下同）系统规划建设托育服务体系。城市政府提供全方位政策支持清单。企业（含企业、事业单位、社会组织等，下同）提供普惠托育服务清单，向社会公开、接受监督。城企双方签订合作协议，扩大普惠性托育服务有效供给，满足家庭多层次、多样化托育服务需求，增强人民群众获得感、幸福感和安全感。

　　**二、**基本原则
　　（一）普惠导向。支持面向社会大众的普惠性托育服务项目，为婴幼儿家庭提供质量有保障、价格可承受、方便可及的托育服务。
　　（二）自愿参加。鼓励有积极性的城市自愿申报，鼓励信用好、有投资意愿的企业按照给定条件自愿申请，鼓励有资质的金融机构自愿参与。对企事业单位、营利非营利机构、国企民企、内资外资均一视同仁。
　　（三）竞争择优。优先考虑规划科学、基础扎实、政策力度大的城市；优先支持诚实守信、项目优质、专业能力强的企业；优先选择融资成本低、服务质量好的金融机构。
　　（四）安全规范。牢固树立安全意识，把婴幼儿安全和健康摆在最为突出的位置，严格执行相关法律法规。明确责任主体，做到建设和运营规范，监管到位，确保项目安全运行。

　　**三、**工作目标
　　建成一批具有带动效应、承担一定指导功能的示范性托育服务机构，社区托育服务骨干网基本完善，普惠性托位数量大幅增加，服务内容不断丰富，服务质量明显提升，对专业人才队伍建设支撑更加有力，对家庭科学养育指导能力持续增强，更多更好惠及婴幼儿家庭。

　　**四、**中央预算内投资支持方式及内容
　　国家通过中央预算内投资，重点支持以下两类托育服务设施建设。
　　一是承担一定指导功能的示范性托育服务机构。示范性托育服务机构具备托育服务功能，设置一定规模的普惠性托位，并提供托育从业人员培训、托育机构管理咨询、家庭养育指导和社区亲子服务等服务。示范性托育服务机构可以选址新建，也可利用早期教育指导中心、妇女儿童活动中心、妇女儿童之家、家庭教育指导服务中心、学前教育机构、计划生育服务机构、月子中心、家政服务公司等资源改扩建（含改建、扩建，下同）。
　　二是社区托育服务设施。通过新建、改扩建，支持一批嵌入式、分布式、连锁化、专业化的社区托育服务设施建设，提供全日托、半日托、计时托、临时托等多样化的普惠托育服务。支持在新建居住区等配建托育服务设施；支持在老城区和已建成居住区新建、改扩建托育服务设施；支持学前教育机构等通过新建、改扩建等方式提供托育服务。鼓励托育服务设施与社区服务中心（站）及社区文化、体育、养老等设施共建共享。
　　政府机关、企事业单位利用自有土地或设施新建、改扩建托育服务设施，并对社会开放普惠性托位的，也可纳入以上两类支持范围。

　　**五、**工作任务
　　（一）明确参与主体及责任
　　-- 城市政府负责制定托育服务体系建设规划（方案），研究出台土地、场所、人才培养、财税优惠、包容审慎监管等全方位政策支持清单，提出发展目标并谋划一批普惠性托育服务项目，明确项目类型、规模、投融资方式、服务人群、计划开工时间以及服务要求及监管方式等。
　　-- 企业负责落实投资、明确建设内容及运营方案，并可通过自营、委托运营等方式提供普惠性托育服务。
　　城企双方签订合作协议，城市政府参照《地方政府支持政策清单》（附件1），明确政策支持具体内容，并确保优惠政策落实到位。《地方政府支持政策清单》分为必选项和自选项，必选项是城市申报专项行动的必要条件，自选项是专项行动择优遴选试点城市的重要参考依据，自选政策越多、政策含金量越高的城市优先纳入试点范围。
　　企业要参照《企业责任承诺清单》（附件2），明确普惠性托育服务具体内容，向社会公开、接受监督。参与企业有义务按照要求向政府有关部门报送进展情况。
　　（二）明确参与流程
　　--项目申报。有意愿的城市政府向省级发展改革委报送项目，提交政策承诺函。省级发展改革委牵头会同卫生健康委（或省政府明确的行业主管部门）组织审核后，按程序报送国家发展改革委，并抄送国家卫生健康委。建设项目具体要求按《支持社会力量发展普惠托育服务专项行动项目和资金管理办法（试行）》（附件3）执行。
　　--中央预算内投资适当补助。采用补助的方式，对于承担一定指导功能的示范性托育服务机构、社区托育服务设施，中央预算内投资按每个新增托位给予1万元的补助。
　　--资金下达。地方项目采用切块下达的方式，支持已开工或当年拟开工项目，即“××省××年支持社会力量发展普惠托育服务项目”，将中央预算内投资下达到相关省份，由省份在收到下达通知书20个工作日内将资金分解到具体项目。
　　--项目管理。城市政府对每个项目都要现场调研、查验，确保项目建设保时、保量、保质、保真，实施过程中的重大问题及时报省级和国家发展改革委、卫生健康委。城市政府需于参加专项行动当年年底前，完成本地区托育服务体系建设规划（方案）编制并报国家发展改革委备案。
　　（三）支持措施
　　--金融支持。形成金融机构推荐名单并实行动态管理，与国家发展改革委签订备忘录，对参与专项行动的托育机构提供普惠金融服务。
　　--信用评价。引入第三方信用服务机构，依托全国信用信息共享平台、国家企业信用信息公示系统和地方各级信用信息平台，整合公共信用信息和市场信用信息，对托育服务机构开展公共信用综合评价。推动实施托育服务行业守信联合激励和失信联合惩戒，建立托育服务机构及从业人员“黑名单”制度。
　　--试点示范。2020年开展专项行动试点，参与试点城市要依托示范性托育服务机构和社区托育服务设施建设，充分吸引社会力量广泛参与，强化政策支持和服务监管，扩大托育服务有效供给。在试点基础上，遴选支持社会力量发展普惠托育服务重点联系城市，通过现场经验交流、典型案例征集等形式，及时总结推广典型经验和先进做法。

　　**六、**附则
　　本方案自公布之日起实施，根据实际情况适时调整。
　　附件：1.地方政府支持政策清单
　　2. 企业责任承诺清单
　　3. 支持社会力量发展普惠托育服务专项行动项目和资金管理办法（试行）
　　附件1

　　地方政府支持政策清单

　　严格落实中央支持政策，例如根据《关于养老、托育、家政等社区家庭服务业税收优惠政策的公告》（财政部税务总局发展改革委民政部商务部卫生健康委公告2019年第76号），对社区托育服务落实税收优惠和费用减免政策。同时，在本辖区范围内明确地方具体支持政策如下。

　　**一、**必选项
　　（一）土地、规划政策
　　1. 允许教育、医卫、福利、商服等用地类别用于发展托育服务，纳入国土空间规划和年度用地指标，区分营利性和非营利性，优先安排土地利用计划。
　　2. 对符合《划拨用地目录》的非营利性托育用地，可采取划拨方式予以保障。对不符合《划拨用地目录》的托育用地，以有偿使用方式予以保障，其有偿使用底价按教育、医卫、福利等用地评估价评估后确定。出让方式取得的土地可以抵押，在符合不改变土地用途等相关规定下，若原企业退出，可由其他具备相关资质的托育企业承担。
　　3. 在新建居住区规划、建设托育服务设施及配套安全设施，与住宅同步验收、同步交付使用。
　　4. 创造条件允许在不调整规划的情况下，由企业利用城镇现有闲置且符合卫生、防护等标准的设施进行改造建设，举办托育服务机构。涉及到土地手续的，可先建设后变更土地使用性质。
　　（二）报批建设政策
　　5. 依法简化社区托育服务登记备案程序，建立多部门开办手续一站式办理的绿色通道，切实缩短企业办证时间。
　　6. 对于托育企业开展连锁化、专业化服务的，在协议明确范围内开设单个服务实体，在登记部门实行备案制，不再单独报批，可合并到总公司统一纳税。
　　（三）人才支持政策
　　7. 推进高等院校和职业院校开设托育人才培养专业，培育相关管理、技术技能型应用人才。
　　8. 将托育从业人员列入急需紧缺职业（工种）目录和政府补贴性培训目录，把育婴员、保育员等托育从业人员纳入当地政府职业技能培训计划，按规定落实职业培训补贴、职业技能鉴定补贴。
　　（四）卫生、消防等支持政策
　　9. 卫生健康部门及其医疗、卫生、保健机构对辖区内托育机构进行管理和医疗、儿童保健、膳食营养、疾病防控等技术指导，为托育从业人员培训提供技术支持。托育机构可作为儿科等相关医护人员基层服务定点单位，服务时长作为基层服务时间，在医护人员申报专业技术高级职称时作为评分条件使用。
　　10.做好托育机构消防审批服务，建立工作机制，对试点项目采用一事一议，提高审批效能。
　　（五）普惠托育服务价格
　　11.按照质量有保障、价格可承受、方便可及的普惠性导向，综合考虑当地居民收入水平、服务成本、合理利润等因素，通过市场形成普惠托育服务价格。具备招标条件的，通过招标方式确定价格水平；不具备招标条件的，与企业通过协商确定价格水平。
　　（六）监督管理政策
　　12.建立项目长期跟踪监管机制，原则上要确保支持项目长期可持续运营。因故确需退出的，应由其他托育机构承接。

　　**二、**自选项
　　（一）土地、规划政策
　　1. 提供公租房免费用于发展托育服务。
　　2. 可使用村集体建设用地用于托育机构建设，由企业与村集体约定土地使用和利益分配方案。
　　3.鼓励支持采取政府和社会资本合作（PPP）方式的项目，发展普惠托育服务。
　　4.人员密集地区的国有营业场地优先用于托育机构建设，纳入当地公共资源交易平台，限定租赁用途，以较低的租赁价格提供给托育服务机构，营业场地的租赁期限一般约定在10年及以上。
　　（二）报批建设政策
　　5.对于利用老旧建筑改造为托育设施，采取“一事一议”的方式，简化规划等前期手续，加快办理施工许可证。
　　6. 充分利用社区资源，协调设置室外活动场地。
　　（三）财税补贴政策
　　7. 采取建设补贴、运营补贴或者以奖代补等形式支持普惠性托育机构发展。
　　8. 托育机构用电、用水、用气、用热按居民生活类价格执行；托育机构申请办理电、水、气、热等业务，实行限时办结制度。
　　9. 将托育从业人员相关技能培训项目列入职业技能培训补贴目录。
　　（四）金融支持政策
　　10.协调地方金融机构为试点项目建设创新服务，提供低息贷款。
　　11.将托育服务项目纳入到政府出资或参股的融资担保机构的支持范围。
　　12.鼓励商业保险机构开发托育机构综合责任保险。
　　（五）其他支持政策
　　13.鼓励中心城区范围内的商务楼宇综合设置普惠托育机构，并合理延展租赁期，保障托育机构可持续运行。
　　14.完善运营补贴、生均经费等优惠政策，支持存量托育机构发展普惠托育服务。
　　15.有助于普惠托育发展的其他政策。例如，鼓励发展婴幼儿储蓄等支持婴幼儿健康成长的金融创新等。（请注明）
　　附件2

　　企业责任承诺清单

　　**一、**必选项
　　1. 严格执行托育管理的相关政策和规范。
　　2. 承担一定指导功能的示范性托育服务机构科学测算年度培训任务、社区亲子活动和家庭托育指导任务，制定年度计划，并接受相关部门考核。
　　3. 确保将政府提供的托育用地或用房用于托育机构建设，不用于其他用途。
　　4. 按照质量有保障、价格可承受、方便可及的普惠性导向，综合考虑当地居民收入水平、服务成本、合理利润等因素，通过市场形成普惠托育服务价格。招标情况下，通过投标竞争方式确定价格水平；非招标情况下，与城市政府通过协商确定价格水平。
　　5. 严格按照相关政策要求使用补贴，确保各类补贴政策精准执行。
　　6. 建立安全制度，确保人身、食品、消防安全。
　　7. 强化诚信建设。
　　8. 加强从业人员管理，建立从业人员档案管理体系，完善从业人员评价制度，加强从业人员职业道德教育。
　　9. 符合《托育机构设置标准（试行）》《托育机构管理规范（试行）》要求。
　　10.建立本机构托育服务日常管理制度和工作规范，提高服务能力，保障服务质量。

　　**二、**自选项
　　1. 承担一定指导功能的示范性托育服务机构协助地方政府建立家庭保育支持体系。
　　2. 丰富服务内容，根据需求提供个性化、多样化的托育服务等。
　　3. 为社区提供婴幼儿健康营养讲座、科学照护讲座等公益性服务。
　　4. 针对有特殊需求的婴幼儿及其家庭，提供个性化托育服务或家庭托育咨询。
　　5. 遵循婴幼儿成长特点和规律，促进婴幼儿在身体发育、动作、语言、认知、情感与社会性等方面的全面发展。
　　6. 建立从业人员职业上升通道。
　　7. 企业可采取的有助于促进普惠托育发展的其他措施（请注明）。
　　附件3

　　支持社会力量发展普惠托育服务专项行动项目和资金管理办法（试行）

　第一章　总则

　　**第一条**　为规范支持社会力量发展普惠托育服务专项行动实施方案（以下简称“实施方案”）项目和投资管理，加强组织实施，提高中央预算内投资使用效益，依据国家有关法律法规和《[中央预算内投资补助和贴息项目管理办法](https://www.pkulaw.com/chl/f0158d3f374b9244bdfb.html)》（国家发展和改革委员会令第45号，以下简称“45号令”）等要求，制定本办法。

　　**第二条**　根据实施方案，2020年开展专项行动试点，选择积极性高、前期基础扎实的城市择优实施项目建设，并可根据中央预算内投资安排情况和项目执行情况展期实施。

　　**第三条**　实施方案覆盖范围包括各省、自治区、直辖市及计划单列市，新疆生产建设兵团，黑龙江省农垦总局（以下简称“各省”）。

　　**第四条**　实施方案旨在增加普惠性托育服务供给，为婴幼儿家庭提供质量有保障、价格可承受、方便可及的托育服务，逐步形成布局合理、设施完善、服务便捷、保障有力的普惠托育服务体系。

　第二章　管理职责和工作程序

　　**第五条**　国家发展改革委会同国家卫生健康委负责编制实施方案、制定项目和资金管理办法、组织实施和监督检查。

　　**第六条**　城市政府根据实施方案要求，进行项目储备，明确项目类型、规模、投融资方式、服务人群及计划开工时间等，并将符合条件的项目纳入三年滚动投资计划，列入国家重大建设项目库。

　　**第七条**　城市政府根据托育服务体系建设整体规划、合作协议向省级发展改革部门报送项目，经省级发展改革部门牵头会同卫生健康部门（或省级政府明确的行业主管部门）审核后向国家发展改革委报送中央预算内投资年度资金申请文件和项目；国家发展改革委按建设程序下达年度投资计划。

　　**第八条**　中央预算内投资采取切块下达的方式，由国家发展改革委下达到省级发展改革部门。投资计划一经下达，原则上不再调整。执行过程中确需调整的，由各省发展改革部门做出调整决定并报国家发展改革委备案。

　　**第九条**　各省发展改革部门是资金分解下达的责任主体，城市政府是项目申报、实施过程监管的责任主体。各省发展改革部门将中央预算内资金安排到具体项目的权力不得下放，需在收文后20个工作日内将资金分解到具体项目，分解下达投资计划时要明确项目建设地点、建设规模、建设工期及配套投资比例和数额，并向国家发展改革委报送备案文件；城市政府要加强对项目推进全过程监督与管理。

　第三章　支持范围和遴选方式

　　**第十条**　通过中央预算内投资，重点支持两类托育服务设施建设项目：
　　一是承担一定指导功能的示范性托育服务机构。示范性托育服务机构具备托育服务功能，设置一定规模的普惠性托位，并提供托育从业人员培训、托育机构管理咨询、家庭养育指导和社区亲子服务等。示范性托育服务机构可以选址新建，也可利用早期教育指导中心、妇女儿童活动中心、妇女儿童之家、家庭教育指导服务中心、学前教育机构、计划生育服务机构、月子中心、家政服务公司等资源改扩建（含改建、扩建，下同）。
　　二是社区托育服务设施。通过新建、改扩建，支持一批嵌入式、分布式、连锁化、专业化的社区托育服务设施建设，提供全日托、半日托、计时托、临时托等多样化的普惠托育服务。支持在新建居住区等配建托育服务设施；支持在老城区和已建成居住区新建、改扩建托育服务设施；支持学前教育机构等通过新建、改扩建等方式提供托育服务。鼓励托育服务设施与社区服务中心（站）及社区文化、体育、养老等设施共建共享。
　　政府机关、企事业单位利用自有土地或设施新建、改扩建托育服务设施，并对社会开放普惠性托位的，也可纳入以上两类支持范围。

　　**第十一条**　项目遴选基本条件包括：
　　（一）项目要符合国土空间规划及社会事业发展规划要求，与当地人口、土地、环境、交通等实际状况相适宜，选址布局科学合理。
　　（二）项目建设必须符合国家有关法律法规要求，执行环境保护、节约土地、安全管理、节约能源等有关方面的规定。
　　（三）项目要符合45号令以及中央预算内投资管理的有关规定，已经开工建设或前期工作准备成熟，投资计划下达后当年即可开工建设，配套资金足额落实。
　　（四）项目要按照《国家发展改革委办公厅关于使用国家重大建设项目库加强项目储备编制三年滚动投资计划有关问题的通知》（发改办投资〔2015〕2942号）要求，做好与三年滚动投资计划的衔接，并录入重大建设项目库。

　第四章　项目建设要求和补助标准

　　**第十二条**　对于承担一定指导功能的示范性托育服务机构和社区托育服务设施，中央预算内投资按每个新增托位给予1万元的补助。
　　托育服务设施建设的功能布局、参数设置等应按照《托育机构设置标准（试行）》和抗震、防火、防洪等国家相关标准的规定执行，并可参考《托儿所、幼儿园建筑设计规范》（JGJ39）的规定。

　第五章　资金安排原则和使用管理

　　**第十三条**　各省发展改革委要根据项目前期深度，优先遴选符合支持条件且前期成熟的项目纳入中央预算内投资支持范围。按照在建和开工前阶段（包括施工许可、施工合同签订、施工招投标等）、规划许可阶段（包括建设工程规划许可、建设用地规划许可、确定规划设计方案等）以及取得国有建设用地使用权阶段（包括土地出让合同或划拨决定书等）梯次对项目排序。具体项目申报程序，区分不同建设主体，按照原有规定管理。国家发展改革委按照项目成熟度，统筹考虑区域、人口等因素进行平衡。

　　**第十四条**　中央预算内投资实行专款专户管理，防止转移、侵占或者挪用，确保中央预算内资金的合理使用和项目建设顺利实施。

　　**第十五条**　中央预算内投资用于计划新开工或者在建项目，不得用于已完工项目。同一项目不得重复申请不同专项资金。

　第六章　监督管理

　　**第十六条**　城市政府要对项目资金使用、实施效果负总责。应当加强项目的监督管理，采取事前、事中、事后相结合，日常监督和专项监督相结合的方式，对项目建设资金使用实施全过程监督管理。

　　**第十七条**　建设项目要严格执行项目法人责任制、招标投标制、工程监理制和合同管理制等建设管理法规，项目设计单位和施工单位必须具有相应资质，做到公平、公正、公开、透明。各地要加强项目竣工验收，适时将年度投资计划、竣工验收情况上报国家发展改革委。

　　**第十八条**　各地要定期对项目质量、进度、资金使用情况等进行监督检查，及时解决建设过程中存在的问题，确保建设项目保质保量如期完成。国家发展改革委会同国家卫生健康委适时对普惠托育项目执行情况进行监督检查。

　　**第十九条**　各地要通过投资项目在线审批监管平台（重大建设项目库模块）于每月10日前对项目年度投资计划执行情况进行调度，及时填报项目开工情况、投资完成情况、工程形象进度等数据。

　　**第二十条**　项目申报单位（省级发展改革部门）有下列行为之一的，国家发展改革委可根据情节，在一定时期和范围内不再受理其报送的资金申请报告，或者核减、收回或停止拨付中央预算内投资并予以通报。
　　（一）指令或授意项目单位提供虚假情况、骗取投资补助资金的；
　　（二）对申报项目审核不严，造成投资补助资金损失的；
　　（三）对于切块下达的年度投资计划分解和安排出现拖延或严重失误的；
　　（四）所在地区项目存在较多问题且督促整改不到位的；
　　（五）未按要求通过线审批监管平台报告相关项目信息的；
　　（六）其他违反国家法律法规的行为。

　　**第二十一条**　参与城市有下列行为之一的，国家发展改革委可根据情节，取消其专项行动试点城市资格，并在一定时期内不接受其参与申请。
　　（一）未按时制定托育服务体系建设整体规划的；
　　（二）未按时提交政策支持承诺函或违反承诺的。

　　**第二十二条**　参与企业有下列行为之一的，国家和省级发展改革部门可责令其限期整改；拒不整改或整改后仍不符合要求的，应当核减、收回或停止拨付投资补助资金，暂停受理其申报项目申请，将相关信息纳入全国信用信息共享平台和在国家企业信用信息公示系统依法公示，并视情节轻重提请或移交有关机关依法追究有关责任人的责任。
　　（一）提供虚假情况，骗取投资补助资金的；
　　（二）转移、侵占或者挪用投资补助资金的；
　　（三）擅自改变主要建设内容和建设标准的；
　　（四）项目建设规模、标准和内容发生较大变化而不及时报告的；
　　（五）无正当理由未及时建设实施的；
　　（六）拒不接受依法进行的稽察或者监督检查的；
　　（七）未按要求通过在线平台报告相关项目信息的；
　　（八）其他违反国家法律法规的行为。

　第七章　附则

　　**第二十三条**　本办法由国家发展改革委负责解释。

　　**第二十四条**　本办法自发布之日起施行，有效期为2年。

© 北大法宝：（[www.pkulaw.com](https://www.pkulaw.com/)) 专业提供法律信息、法学知识和法律软件领域各类解决方案。北大法宝为您提供丰富的参考资料，正式引用法规条文时请与标准文本核对。
欢迎查看所有[产品和服务](https://www.pkulaw.com/helps/2.html)。法宝快讯：[如何快速找到您需要的检索结果？](https://www.pkulaw.com/helps/70.html)[法宝](https://www.pkulaw.com/helps/69.html)V6有何新特色？

扫描二维码阅读原文

**原文链接：**<https://www.pkulaw.com/chl/e244b92b13151e10bdfb.html>
